# Polymorphic segmental duplications at 8p23.1 challenge the determination of individual defensin gene repertoires and the assembly of a contiguous human reference sequence

**DOI:** 10.1186/1471-2164-5-92

**Published:** 2004-12-10

**Authors:** Stefan Taudien, Petra Galgoczy, Klaus Huse, Kathrin Reichwald, Markus Schilhabel, Karol Szafranski, Atsushi Shimizu, Shuichi Asakawa, Adam Frankish, Ivan F Loncarevic, Nobuyoshi Shimizu, Roman Siddiqui, Matthias Platzer

**Affiliations:** 1 Genomanalyse, Institut für Molekulare Biotechnologie, Beutenbergstr. 11, D-07745 Jena, Germany; 2 Keio University School of Medicine, 35 Shinanomachi, Shinjuku-ku, Tokyo 160-8582, Japan; 3 Wellcome Trust Sanger Institute, Hinxton Cambridge CB10 1SA, UK; 4 Institut für Humangenetik und Anthropologie, Friedrich-Schiller-Universität Jena, Kollegiengasse 10, D-07743 Jena, Germany

## Abstract

**Background:**

Defensins are important components of innate immunity to combat bacterial and viral infections, and can even elicit antitumor responses. Clusters of defensin (DEF) genes are located in a 2 Mb range of the human chromosome 8p23.1. This DEF locus, however, represents one of the regions in the euchromatic part of the final human genome sequence which contains segmental duplications, and recalcitrant gaps indicating high structural dynamics.

**Results:**

We find that inter- and intraindividual genetic variations within this locus prevent a correct automatic assembly of the human reference genome (NCBI Build 34) which currently even contains misassemblies. Manual clone-by-clone alignment and gene annotation as well as repeat and SNP/haplotype analyses result in an alternative alignment significantly improving the DEF locus representation. Our assembly better reflects the experimentally verified variability of DEF gene and DEF cluster copy numbers. It contains an additional DEF cluster which we propose to reside between two already known clusters. Furthermore, manual annotation revealed a novel DEF gene and several pseudogenes expanding the hitherto known DEF repertoire. Analyses of BAC and working draft sequences of the chimpanzee indicates that its DEF region is also complex as in humans and DEF genes and a cluster are multiplied. Comparative analysis of human and chimpanzee DEF genes identified differences affecting the protein structure. Whether this might contribute to differences in disease susceptibility between man and ape remains to be solved. For the determination of individual DEF gene repertoires we provide a molecular approach based on DEF haplotypes.

**Conclusions:**

Complexity and variability seem to be essential genomic features of the human DEF locus at 8p23.1 and provides an ongoing challenge for the best possible representation in the human reference sequence. Dissection of paralogous sequence variations, duplicon SNPs ans multisite variations as well as haplotypes by sequencing based methods is the way for future studies of interindividual DEF locus variability and its disease association.

## Background

Despite the tremendous efforts and successful completion of the Human Genome Project by April 14^th ^2003, a set of recalcitrant gaps remain in the euchromatic part of the final human genome sequence. One obvious reason for these gaps is that the appropriate regions are enriched in sequences that are not tolerated by the cloning systems. The second possibility is that even if clones are available and amenable for sequencing, their sequences cannot be unambiguously aligned due to gap flanking segmental duplications. Generally, those duplicons are defined by >90% sequence identity and lengths of >1 kb and about 87% of all human ones are longer than 50 kb [1]. In these regions with nucleotide identities up to >99% over several kb it is nearly impossible to decide whether very similar sequences represent distinct loci or different alleles of a single locus. Here, sequencing of a single chromosomal haplotype is a straightforward approach to achieve a „consistent“ assembly. It was successfully applied to decipher intrachromosomal duplications of the human Y [2]. If, however, duplications are located on autosomes and their copy numbers vary interindividually, as shown for regions in 15q11-q13 [3], the situation becomes even more complicated and requires the extra effort of resolving haplotype differences that result from the diploid nature of the underlying BAC library. In the Williams-Beuren syndrome (WBS) region on human chromosome 7, only extensive redundant sequencing from a single BAC library led to a representative sequence [4]. Alternatively, monospermic complete hydatidiform moles [5,6] and hamster somatic cell hybrids [7] provide access to fully homozygous genomes or individual autosomes, respectively.

It is a fact that structural variations between chromosomal haplotypes complicate the sequence assembly and lead to the formation of *de facto *gaps [1,8]. The more haplotypes are represented by BAC clones, the more *de facto *gaps may be formed. In the case of unresolved segmental duplications, usually a large number of clones has been sequenced with high accuracy [9] and the clone coverage of the loci is well above-average of the entire human genome. However, no contiguous tiling path can be build and gaps remain. Nevertheless, the available data are an invaluable resource for the investigation of individual genetic variations in duplicated regions and of their association with diseases.

**Figure 1:**
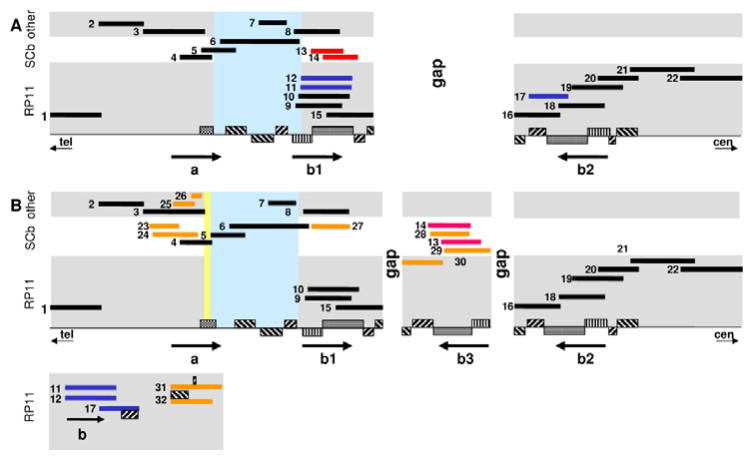
**Alternative alignments of the 8p23.1 DEF locus. **(*A*) July 2003 UCSC version hg16 [10] chr8:6,258,283-8,262,034. Only finished clones are shown and arranged by libraries, which are indicated by background colors: RP11 = gray, bottom; SCb = white, middle; other (CTB, CTD, GS, RP13) = gray, top. Defensin gene clusters are shown as arrows, repeat blocks are indicated as striped boxes, (+) strand is above the black line, (-) strand is below the black line, same stripe patterns indicate similar structures. The light blue background indicates the distal repeat region for chromosomal rearrangements [16]. (*B*) Revised alignment of the 8p23.1 DEF locus, containing an additional 360-kb-contig and five clones which cannot be aligned; colors: black = aligned as in Fig. 1A; orange = clones not present in the UCSC browser; red = clones with different positions in both alignments, blue = clones presented in the UCSC browser but excluded in the revised assembly. The yellow vertical bar in DEF a illustrates the widening of the *DEF cluster a *as a result of the alternative alignment of [GenBank:AF200455] / [GenBank:AF238378] (see text). Clone (number) GenBank accession.version / library: (1) [GenBank:AC018398] / RP11, (2) [GenBank:AF287957] / CTD, (3) [GenBank:AF233439] / GS, CTD, (4) [GenBank:AF200455] / SCb, (5) [GenBank:AF238378] / SCb, (6) [GenBank:AF228730] / SCb, CTB, (7) [GenBank:AF215847] / CTB, (8) [GenBank:AC130339] / RP13, (9) [GenBank:AC130360] / RP11, (10) [GenBank:AC130367] / RP11, (11) [GenBank:AC134395] / RP11, (12) [GenBank:AC134683] / RP11, (13) [GenBank:AC285443] / SCb, (14) [GenBank:AC202031] / SCb, (15) [GenBank:AC134684] / RP11, (16) [GenBank:AC084121] / RP11, (17) [GenBank:AC144950] / RP11, (18) [GenBank:AC130365] / RP11, (19) [GenBank:AC131269] / RP11, (20) [GenBank:AC105233] / RP11, (21) [GenBank:AC068020] / RP11, (22) [GenBank:AC068353] / RP11, (23) [GenBank:AF298854] / SCb, (24) [GenBank:AF205406] / SCb, (25) [GenBank:AF314060] / GS, (26) [GenBank:AF314059] / SCb, (27) [GenBank:AF252831] / SCb, (28) [GenBank:AF189745] / SCb, (29) [GenBank:AF252830] / SCb, (30) [GenBank:AC148106] / RP11, (31) [GenBank:AC105214] / RP11, (32) [GenBank:AC092766] / RP11

**Figure 2:**
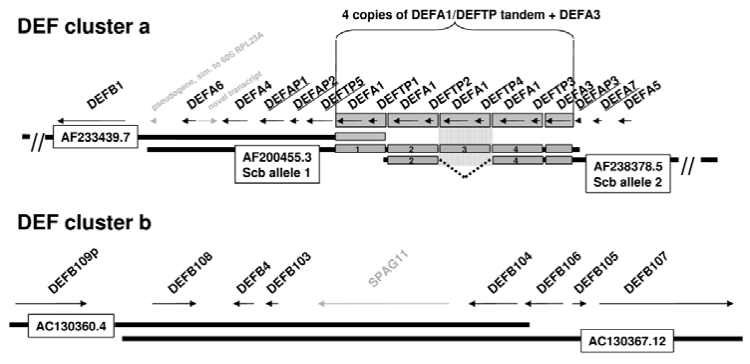
**Genes and pseudogenes in *DEF clusters a *and *b*. **Names correspond to the Vertebrate Genome Annotation, intergenic distances are scaled 1:10. Defensin and defensin like genes and pseudogenes are written in black, novel defensin genes and pseudogenes are underlined, other genes / transcripts are indicated in gray. ***DEF cluster a*: **The presence of four copies of the *DEFA1/DEFTP *tandem and the *DEFA3 *gene in [GenBank:AF200455] requires the illustrated clone alignment, resulting in a "widening" of the hg16 assembly, pictured by the striped gray box (corresponding to the yellow bar in Fig.1). Analysis of the intergenic distances (data not shown) suggests, that [GenBank:AF238378] harbors copies 2 and 4 of the *DEFA1/DEFTP *tandem whereas copy 3 is missing (dotted line). Since both clones are derived from the same library (SCb), either copy 3 is lost during the cloning process or the clones represent different alleles. ***DEF cluster b*: **The *DEF cluster b *is illustrated in the orientation of *DEF cluster b1*.

One of those complex regions is located in 8p23.1 at 6.3 – 8.3 Mb of the July 2003 human reference sequence (NCBI Build 34; UCSC version hg16, Fig. 1A). In the Golden Path assembly [10], there are 22 finished clones from five different libraries and 20 working draft or predraft clones (<4x coverage shotgun; four different libraries) grouped on both sides of a recalcitrant gap at 7.5 Mb. Another 10 finished clones from four different libraries are not included in the hg16 assembly but map to the 8p23.1 locus. Several attempts to close this gap have failed due to the highly repetitive structure of the flanking sequences. The gap flanking regions harbor defensin (DEF) genes, encoding a group of small cationic peptides with characteristic three intramolecular disulfide bonds. These peptides play a prominent role in innate immunity to defend bacterial and viral infections in animals, plants and insects [11]. Furthermore, in humans, loss or down regulation of DEF genes is shown to be related with cancer, such as renal cell carcinoma [12-14], prostate cancer [14] and bladder tumors [15]. Two different DEF gene clusters can be distinguished: *DEF cluster a *contains the genes *DEFB1*, *DEFA6*, *DEFA4*, *DEFA1*, *DEFT1*, *DEFA3 *and *DEFA5*; *DEF cluster b *comprises the genes *DEFB109p*, *DEFB108*, *DEFB4*, *DEFB103*, *SPAG11*, *DEFB104*, *DEFB106*, *DEFB105*, and *DEFB107 *(Fig. 2). *DEF cluster b *is duplicated in reverse complementary orientation on either side of the gap, forming the distal *cluster b1 *and the proximal *cluster b2*.

Interestingly, the DEF cluster region was identified as the distal breakpoint (REPD) of a 4.7-Mb segment inversion, identified as a common polymorphism with frequencies of 39% and 26% in the Japanese population and in Europeans, respectively [16-18]. Although the inversion itself apparently do not have any pathological effects, in heterozygous female carriers unequal recombinations can occur, leading to three macrorearrangements – inv dup del (8p); +der(8)(8p23.1pter) and del (8)(p23.1p23.2) – related to severe disease phenotypes. The fact that low copy repeats (LCR) flanking the DEF clusters represent the essential sites for such recombination events is a strong argument to resolve the structure of the LCRs themselves as well as the genomic organization of the entire region. Also, genes of both *DEF cluster a *and *b *vary interindividually in their copy numbers. This was shown by somatic cell hybrid mapping for *DEFA1*, *DEFA3 *(*DEF cluster a*; 2–3 copies each) [19] and by a combination of multiplex amplifiable probe hybridisation and semiquantitative fluorescence in situ hybridization for *DEFB4*, *DEFB103*, *DEFB104 *(*DEF cluster b*; 2–12 copies each) [20]. It is generally assumed that this variability crucially contributes to the differences in the innate immunity network between individuals and influences predisposition and susceptibility for diseases.

The polymorphic nature of this locus suggested to us that the pool of clones presented in the hg16 assembly should be aligned in a different way. Our alternative assembly creates more DEF cluster copies and better reflects the individual variability of the locus. In addition, comparative sequence analysis of DEF genes in our closest relative, the chimpanzee (*Pan troglodytes*), both revealed the differences in the defensin protein panel of both species and showed that DEF clusters are also multiplied in the ape.

Moreover, extraction of single nucleotide polymorphisms (SNPs) from overlapping regions of clones harboring DEF genes provided haplotypes which were analyzed for their ratio in individuals and used for the determination of individual gene copy numbers.

## Results

### Revision of the hg16 assembly

In the framework of the International Human Genome Sequencing Consortium we sequenced 19 out of 32 BAC clones mapping to the 8p23.1 DEF region (for linking clone numbers with accession numbers see legend of Fig. 1; accession numbers and corresponding clone names are given in Additional file 1). In addition to the existing clone alignments of *DEF clusters b1 *and *b2*, in our sequence assembly a consistent 360-kb-contig comprising five clones was built that also contains a *DEF cluster b*. Interestingly, this additional contig could neither be joined unambiguously to *cluster b1 *nor to *cluster b2*. This is in contradiction to the hg16 assembly where clones 13 and 14 of the novel contig are positioned in DEF *cluster b1*. This disagreement prompted us to evaluate carefully the alignment of the entire region. For this, we used all 32 finished clones which map in the region. To circumvent the problems of an automatic assembly in repeat rich, duplicated regions, the clones were manually joined according to the following criteria: only single base exchanges and insertions/deletions in repeat stretches were tolerated and joins were not performed if the total ratio of single base differences in the overlapping clone portions was >0.8%.

The result is an alternative alignment shown in Fig. 1B, which differs from the hg16 assembly in three major points and is supported by a detailed repeat and SNP/ haplotype analysis:

(1) Clones 23–27 not present in the hg16 assembly were located distally of the gap, whereas clones 11, 12 and 17 were excluded since they cannot be aligned according to our criteria. Clones 13 and 14 were moved to the new 360-kb-contig (see below).

(2) In the framework of the human and vertebrate analysis and annotation initiative (HAVANA) [21], where gene structures are annotated on the basis of human interpretation of combined supportive evidence generated during sequence analysis, we manually annotated seven DEF gene containing clones, five of them located in *DEF cluster a*. We found that clone 4 contains four copies of a *DEFA1 */ *DEFTP *tandem and one copy of *DEFA3*, whereas clone 5 harbors only two *DEFA1 */ *DEFTP *tandems and the *DEFA3 *gene (Fig. 2). Consequently, in our assembly, clone 4 is aligned to clone 5 in a way that results in four copies of the *DEFA1*/*DEFTP *tandem instead of three copies in the hg16 assembly. Thus, this region will be "widened" by shifting all proximally adjacent clones for 23,270 bp. Analysis of the intergenic distances between the DEF genes suggests that clone 5 harbors copies 2 and 4 of the four *DEFA1 */ *DEFTP *tandems. Furthermore, hitherto undiscovered, additional DEF genes and pseudogenes could be annotated: clones 3–5 harbor the *DEFA7 *gene [GenBank:A98570], [GenBank:A98571] coding for a novel protein, similar to *DEFA4*, as well as pseudogenes *DEFAP1*, *DEFAP2 *and *DEFAP3 *similar to DEFA (Fig. 2). Our annotation of the entire DEF cluster region is submitted to the Vertebrate Genome Annotation database [22].

(3) Our assembly of the 32 finished clones mapping to the locus created an additional 360-kb-contig which consists of five clones representing an additional, third DEF cluster copy named b3. Since the repeat in clone 30 is located on the (-)-strand, the contig cannot be joined to any of the repeats at either side of the gap but instead has to be located within the gap in unknown orientation.

### Low copy repeat analysis

**Figure 3:**
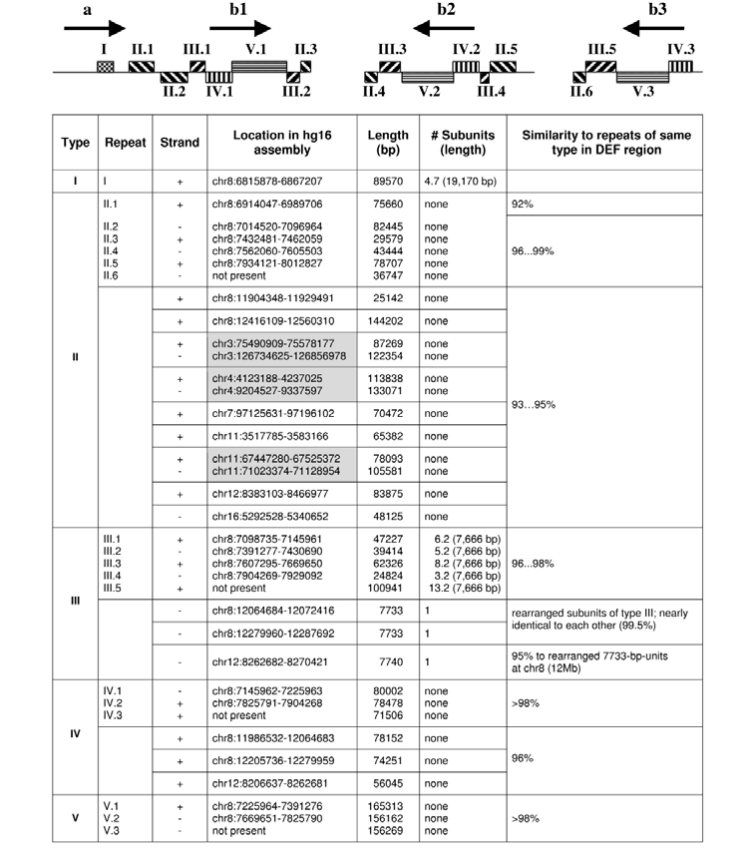
**DEF gene cluster flanking repeat blocks in 8p23.1 and their paralogs on other genomic loci. **Repeats and repeat clusters are drawn as striped bars as in Fig. 1. Inverted repeat pairs of the paralogs are highlighted in gray.

The DEF locus contains several genomewide low copy repeats (LCR) (Fig. 3). The repeats form clusters of up to 165 kb length and paralogs exist on other chromosomes as well as on chromosome 8 at about 12 Mb. *In silico *identification of these paralogs was in good accordance with fluorescence in situ hybridization (FISH) experiments using clones from the 8p23.1 DEF locus as probes (Additional files 3 and 4). Five types of LCRs can be distinguished:

#### Type I

The 89.6 kb cluster consists of 4.7 subunits of a 19.2 kb repeat. Each complete subunit contains *DEFA1 *and *DEFTP*. The incomplete fifth copy harbors *DEFA3*. The content of interspersed repeats is low (20%); the nucleotide identity between the subunits is 99%. The repeat subunits of cluster I are unique to this locus and do not show significant homologies to other regions of the human genome.

#### Type II

Repeats of type II flank *DEF clusters b*, do not show substructures and do not contain genes. In contrast to repeat units of type I they differ in length and orientation, contain inversions and show a high degree of interspersed repeats (55%). The considerable length differences are caused by an initial partial duplication and/or subsequent deletions. The nucleotide identity of the repeats is 96–99% except for II.1, located adjacent to *DEF cluster a*, which exhibits an identity of ~92% to repeats II.2-6.

Blat search of the longest repeat (II.2, 82 kb) revealed that repeats of type II are also present at about 12 Mb on chromosome 8p23.1 as well as on other human chromosomes, e.g. 3, 4, 7, 11, 12 and 16. The paralogs vary in length (25–133 kb) and show nucleotide identities of 93–95% compared to II.2 on 8p23.1. On chromosomes 3, 4 and 11, they are arranged as inverted repeats. The distances in between "repeat pairs" vary remarkably: Whereas in 11q13.2-11q13.4 and 4p16.2-16.1 the parts are separated by 4 and 5 Mb, respectively, the repeats on chromosome 3 are located in 3p12.3 and 3q13.31, framing 51 Mb on both arms of the chromosome.

#### Type III

Located adjacent to *DEF clusters b*, they differ in length and orientation and consist of various numbers of 7.7 kb-subunits showing a low content of interspersed repeats (17%). The nucleotide identity of two subunits of a single type III cluster is >99%, whereas the identity of two subunits of different clusters is in the range of 96–98%. Each of the 7.7 kb-subunits harbors one copy of a gene for the hypothetical protein FLJ10408 [GenBank:NM_018088]. The coding sequences of the gene copies differ and in some cases the reading frame contains premature termination codons. Blat search of the type III repeat subunit revealed three paralogs in the human genome. Two of these are located on chromosome 8p23.1 at about 12 Mb (flanked by repeats of type II), the third at 12p13.31. The paralogs are slightly rearranged in comparison to the subunits of III.1-5.

#### Type IV

Repeats of this type partially cover the *DEF clusters b *(containing *DEFB109P *and *DEFB108*, Fig. 2) and do not show substructures. The content of interspersed repeats is about 30%; the nucleotide identity between repeats IV.1-3 is >98%. Paralogs of these repeats exist on chromosome 8 at about 12 Mb and on chromosome 12 with identities of about 96% to IV1-3.

#### Type V

These repeats are unique to the 8p23.1 DEF locus, show no substructures and contain 35–38% interspersed repeats as well as the major part of the *DEF cluster b *genes (all genes downstream of *DEFB108*, Fig. 2). The nucleotide identities between V.1, V.2 and V.3 are >98%.

### SNPs and haplotypes

We manually inspected seven clones covering *DEF cluster a *and 16 clones covering *DEF clusters b1*, *b2 *and *b3 *for SNPs in exons and introns of all DEF genes. In total we found 270 overlap SNPs: 25 are located in coding sequences, comprising 16 nonsynonymous and nine synonymous changes. 36 SNPs were identified in untranslated regions, and 209 are located in introns. With respect to the coding SNPs in *DEF clusters b *and regarding 11 of 16 clones, six distinct haplotypes H1-6 can be defined (Table 1). One clone each supports haplotypes H3, H4 and H6, whereas haplotypes H1, H2 and H5 are found in either two or three clones. The remaining five clones harbor only parts of DEF genes rendering the unambiguous identification of coding SNP based haplotypes impossible. Examination of all SNPs leading to amino acid (aa) changes in defensins indicates that diversity in the peptides is not restricted to residues outside and in between the cystein motif, but also occurs in the vicinity of cysteins, or even a cystein itself is changed (rs1800968 in *DEFB1*; C67S; data not shown).

**Table 1:** SNPs and haplotypes H1-H6 extracted from *DEF cluster b *covering clones

**Gene**	**Pos. mRNA^1^**	**Haplotypes^2^**						**Change**
		**H1**	**H2**	**H3**	**H4**	**H5**	**H6**	
DEFB107	13	G	/	/	T	G	T	F5V
DEFB105	107	C	/	C	T	C	C	P36L
DEFB106	125	T	/	C	T	T	T	Silent
DEFB104	42	A	A	A	A	A	G	V10I
DEFB4	78	T	C	C	C	C	C	Silent
	120	T	T	T	T	C	T	Silent
	275	C	C	C	C	T	C	3'UTR
	335	C	C	C	C	G	C	3'UTR
DEFB108	138	A	A	A	A	T	/	Silent
	111	C	C	C	C	T	/	Silent
	97	G	G	A	A	G	/	G33S
DEFB109p	133	A	A	A	/	G	/	V45I
	132	C	C	A	/	A	/	K44N
	119	C	C	G	/	G	/	R40T
	104	T	T	C	/	C	/	S35F
	41	C	C	/	/	A	/	S14ochre

^1 ^mRNA positions are referred to the following human mRNAs: DEFB107 = [GenBank:AY122467]; DEFB105 = [GenBank:NM_152250]; DEFB106 = [GenBank:NM_152251]; DEFB104 = [GenBank:NM_080389], DEFB4 = [GenBank:NM_004942]; DEFB108 = [GenBank:AF540980]; DEFB109p = [GenBank:AF540981].

^2 ^Haplotypes are derived from the following clones (libraries): H1 = 9, 10 (both RP11); H2 = 8 (RP13), 18, 19 (both RP11); H3 = 11 (RP11); H4 = 12 (RP11); H5 = 13, 14, 29 (all SCb); H6 = 28 (SCb).

### Chimpanzee defensin loci

In order to compare the human chromosome 8p23.1 DEF region to the orthologous locus in our closest relative, we both employed the chimpanzee (*Pan troglodytes*, ptr) whole genome shotgun (WGS) working draft (WD, [23]) and high quality chimpanzee BAC sequences. Close inspection of the chimpanzee WD scaffold 32935 (chain ID 462) revealed all orthologs of the genes in human *DEF cluster a *except *DEFA3*. In contrast to the human organization, *ptrDEFA1 *and *ptrDEFTP *were found as single copies. In order to check whether *ptrDEFA1 *and *ptrDEFTP *are also multiplied in the ape, but misassembled into a single locus, we inspected the NCBI trace archive [24] for chimpanzee WGS sequences covering the *ptrDEFA1 *locus. For a region of about 500 bp spanning exon 1 of *ptrDEFA1*, there are shotgun reads representing six different haplotypes. Since the sequences derived from one chimpanzee this is a clear indication that *ptrDEFA1 *is also multiplied in the ape. No evidence was found for the presence of *ptrDEFA3*. Concerning *DEF cluster b *we encountered the same problem: the cluster is represented only once in the chimpanzee WD (chain ID 900), but trace data inspection indicates the presence of several different haplotypes. Additionally, *ptrDEFB108 *and *ptrDEFB109p *are not covered by any chimpanzee WD sequences. As an alternative to the WD approach, we sequenced for *ptrDEF cluster b *three BAC clones. Examination of SNPs in overlapping regions (104 kb) of the three clones [GenBank:AC150655], [GenBank:AC150656], [GenBank:AC150657] revealed three different haplotypes originating from one chimpanzee. The detected aa changes in human and chimpanzee defensins are illustrated in Additional file 5. Interestingly, in the ptrDEFA5 protein, one of the disulfide bridging cysteins is changed into serine (C54S). In ptrDEFB108 the canonical cystein motif is truncated (R53opal) which suggests that *ptrDEFB108 *is a pseudogene, since also the start codon ATG is changed into GTG.

We also used the ptrDEF sequences for the detection of ancestral alleles of all 19 nonsynonymous and synonymous human DEF coding SNPs (Additional file 2).

### Individual DEF haplotypes and copy numbers

In order to determine individual DEF copy numbers we PCR-amplified a 500 bp fragment of *DEFB104 *which contains 4 SNPs, and a 511 bp region of *DEFB4 *containing 5 SNPs in four individuals (Table 2). Three of the *DEFB4 *SNPs (Table 2, SNPs 5,6 and 7) were previously described in a haplotype study in different ethnic populations [25]. The PCR products were cloned, individual clones were sequenced and respective haplotypes were determined according to the base composition at polymorphic positions. Each individual tested bears between three and four different haplotypes of *DEFB104 *and two to four haplotypes of *DEFB4*. In different individuals the ratios of the single haplotypes vary remarkably. For instance, at *DEFB104 *the haplotype GAGC is found in all four individuals but compared to all other haplotypes at ratios of 2:3 (proband 2) to 1:7 (proband 1). Interestingly, this haplotype is also found in the trace archives of chimpanzee and baboon. Furthermore, ratios of the individual haplotypes of *DEFB104 *as well as of *DEFB4 *indicate different *DEF cluster b *numbers in the four individuals. While proband 3 bears five copies, proband 4 most probably harbors eight copies or multiples thereof.

**Table 2:** Haplotype based estimation of gene and cluster copy numbers

**Haplotypes**	**SNP**^1^					**Proband**			
**DEFB104**	**1**	**2**	**3**	**4**		**1**	**2**	**3**	**4**
1	C	A	A	T		50	19	-	5
2	G	A	A	T		-	-	16	-
3	G	A	G	C		15	18	32	9
4	G	G	G	C		65	10	16	58
5	G	A	A	C		-	-	14	-
	**Ratios of single haplotypes**					**3:1:4**	**2:2:1**	**1:2:1:1**	**1:1:6**
	**Minimal gene copy number**					**8**	**5**	**5**	**8**
**DEFB4**	**5**	**6**	**7**	**8**	**9**	**1**	**2**	**3**	**4**
1	C	C	C	G	A	14	-	8	-
2	C	T	G	G	A	-	17	9	5
3	T	C	C	A	G	8	-	8	-
4	T	C	C	G	G	24	68	11	30
	**Ratios of single haplotypes**					**2:1:3**	**1:4**	**1:1:1:1**	**1:6**
	**Minimal Gene copy number**					**6**	**5**	**4**	**7**
	**Minimal *DEF cluster b *copy number**					**8**	**5**	**5**	**8**

^1 ^SNP1: ss28489415, 2: rs2680507 = rs11774031, 3: ss28489416, 4: rs4259430, 5: rs2740090, 6: rs2740091, 7: rs2737912, 8: rs2737913, 9: rs2737531.

## Discussion

The manual clone-by-clone alignment and gene annotation as well as detailed repeat and SNP/haplotype analyses significantly improved the assembly of the human DEF 8p23.1 locus. Eventhough the revised alignment (Fig. 1B) does not represent a gap-free version of the locus and in fact introduces a second *de facto *gap, it better reflects the region in the sense of a „human genome reference“, since the clones harboring copies of *DEF clusters b *derive from three libraries (RP11, RP13, SCb) and may therefore represent up to five alleles (library RP13 is represented by only one clone). Our assembly also reflects better the diversity of all available sequence data of this chromosomal region: 27 out of 32 finished clones are incorporated into the tiling path. The remaining five clones cannot be included in the assembly according to our quality criteria and therefore must be regarded as parts of additional copies or alleles. Furthermore we point out that the identification of a third copy of the *DEF cluster b *in the 360-kb-contig does not represent an allele of *clusters b1 *or *b2 *derived from an alternative library / donor, since besides four SCb clones one RP11 clone is incorporated. With respect to the RP11 library of which most of the *DEF cluster b *covering clones derive we conclude that at present sequence information of at least five variants of the cluster is available from a single individual: *cluster b1 *(clones 9, 10, 15); *b2 *(clones 16 – 20); *b3 *(clone 30); *b4 *(clone 11) and *b5 *(clone 12). All these results are in agreement with the reported interindividual variability of *DEF cluster b *genes [20].

Alignment and analysis of the intergenic distances of clones 4 and 5 (*DEF cluster a*; Fig. 2) show that clone 5 harbors copies 2 and 4 of the four *DEFA1 */ *DEFTP *tandems present in clone 4. Since both clones derive from the same library (SCb), we conclude that either copy 3 of the tandem was lost during the cloning process of clone 5 or the clones represent two different alleles of the same chromosomal locus. This perfectly agrees with the variation in the copy number of *DEFA1 *reported by Mars [19]. Moreover, the identification of new DEF genes and pseudogenes demonstrates the advantages of a curated manual annotation over automatic approaches.

The LCR analysis (Fig. 3) allows to draw conclusions about the role of these repeats in chromosomal rearrangement processes: The difference in nucleotide identities between LCR II.1 at one hand and II.2-6 on the other hand indicates that repeats II.2-6 might be involved in rearrangement events of *DEF clusters b*, whereas repeat II.1, separating *DEF cluster a *from the clusters *b*, has evolved independently from its paralogs. Aditionally, for repeats on chromosome Y, a similar genomic structure as for inverted LCR type II is described in the literature: a 300-kb inverted repeat flanks a 3.5 Mb region that occurs in opposite orientations in different individuals [2]. This supports the assumption that also type II repeats may be involved in homologous recombination events resulting in chromosomal macrorearrangements including inversions, even pericentromeric ones. In particular, this may hold true for the polymorphic 4.7-Mb inv dup del (8p) reported in the literature [16-18]: LCRs of type II are located on chromosome 8p both at 6.9–8.0 Mb and 11.9–12.6 Mb and therefore separated from each other in a range of 3.9–5.7 Mb. Thus, they can be supposed to be inversion breakpoints in REPP and REPD.

The function of the protein encoded by FLJ10408 located in LCR type III is unknown, but the genomic arrangement facilitates proteome plasticity by multiple copies of the same gene.

SNP detection and correct assignment to regions with segmental duplications is not trivial and hampered by paralogous sequence variations [26], duplicon SNPs and multisite variations [6]. Moreover, there is considerable evidence that gene conversion [27,28] promotes allele plasticity in duplicated regions. This is illustrated by the fact that in the UCSC browser ~2800 SNPs from dbSNP [29] are assigned to *DEF cluster a *(224 kb) and the two *DEF clusters b *(196 kb each) resulting in a SNP density of 1 SNP per 220 bp. Close examination indicates that the SNPs are arbitrarily allocated to the two *DEF cluster b *loci present in the hg16 assembly. Therefore, in such regions, only manual clone-by-clone inspection as performed during our assembly process provides a reliable set of SNPs for the determination of haplotypes. Human SNPs such as rs1800968 in *DEFB1*, affecting cysteins (C67S) might be of functional relevance, since the cystein connectivity is assumed to determine the correct fold of the defensins which is essential to elicit chemotactic responses as shown for DEFB103 [30].

In order to answer the question whether the extraordinary complexity of the DEF locus is human specific, we closely inspected the orthologous region of the chimpanzee WD. In contrast to the human organization, *ptrDEFA1 *and *ptrDEF cluster b *were found as single copies. Familiar with the drawbacks of the WGS automatic assembly [31,32], we suspected these loci are also multiplied in the ape, but assembled wrongly into a single locus due to the high nucleotide identity. In accordance with this assumption, we identified WGS reads representing more than two haplotypes in one chimpanzee. In order to overcome the WD problems we sequenced BAC clones containing *ptrDEF cluster b *according to a high quality standard and conclude that it is at least duplicated. This suggests that the DEF locus of the chimpanzee is probably as complex as in humans.

The exceptional genomic complexity and heterogeneity of the human 8p23.1 DEF locus and the prominent position of defensins in the innate immunity framework raise the question whether individual patterns of haplotypes together with their variable copy number affect the functionality of the defensin system. A similar situation is found for a chemokine gene cluster where an individually variable gene copy number of CCL3-L1 regulates the gene's expression and is supposed to affect the susceptibility to and progression of inflammatory diseases [33]. Systematic typing of physically linked SNPs should allow to detect interindividual differences in haplotypes and locus copy numbers. Sequencing provides a robust method for the determination of haplotypes and their frequencies scalable to large numbers as required for association studies. As outlined above, SNP genotyping in duplicated regions is demanding and in addition to very careful initial data mining and laboratory practice requires methods allowing the quantitative assessment of allele ratios like dynamic allele-specific hybridization [34] and pyrosequencing [35] as well as of copy-number-variation like multiplex ligation-dependent probe amplification [36,37] and representational oligonucleotide microarray analysis [38,39]. In order to differentiate between valid SNPs, duplicon SNPs, paralogous sequence variations and multisite variations, complete hydatidiform moles or haploid genomes have to be included in upstream assay validation [6]. Nevertheless, for highly complex and polymorphic regions the significance of single SNP based assays may be insufficient. As shown in our approach, systematic typing of linked SNPs can overcome this limitation. An estimation of haplotype ratios provides information about the copy number, however, it depends on the number of individual clones analyzed. Our results confirm that the easy-to-handle „classical sequencing approach“ is a valuable tool for the determination of DEF gene variants, DEF haplotypes and DEF cluster copy numbers. More detailed analyses will give a catalog of haplotype combinations associated with different phenotypes and diseases. Finally, the presented work provides a set of SNPs and haplotypes suitable for future studies of interindividual DEF locus variability and its disease association.

## Conclusions

Complexity and variability seem to be essential genomic features of the major human DEF locus and of – yet unknown – functional significance for the innate immunity framework. This is supported by our human-chimpanzee genomic comparison. In conclusion of the presented repeat analyses we propose a model of the repeat and DEF cluster organization (Additional file 6) that is consistent with the available sequence information and explains the observed extensive variability of the locus. Since all proposed structural elements of the highly complex locus are available at least once as finished sequence, no fosmid-end mapping problems have been observed (International Human Genome Sequencing Consortium, unpublished results). This is a strong indication that despite there are at least two *de facto *gaps no essential elements of the DEF locus are missing in the human reference sequence. In comparison to its actual representation, our revised clone alignment clearly better represents the 8p23.1 complexity and improves the human reference sequence as an invaluable resource for the investigation of individual genetic variations. Finally, the presented work provides a set of SNPs and haplotypes as well as a robust sequencing based method suitable for future studies of interindividual DEF locus variability and its disease association.

## Methods

### Alignment revision

Clone alignments were performed using the GAP4 assembly program, version 6 [40], using the sequences of the GenBank versions listed in the legend of Fig. 1 and Additional file 1. Additionally, for all clones sequenced at the IMB Jena the original GAP4 projects including the trace data were used. The clones were joined allowing only single base exchanges and insertions/deletions in repeat stretches. Joins were not performed if the total ratio of single base differences in the overlapping clone portions exceeded 0.8%.

### Repeat analysis

LCRs were identified by application of the Miropeats program [41] to the revised alignment. The repeat sequences were extracted from the revised alignment of joined clones following the positions of the Miropeat's output and checked for their nucleotide identity using sim2 [42] and Blast 2.0 [43,44]. Interspersed repeats in the repeat blocks were identified by RepeatMasker (Version: 20040306-web; [45]. Paralogs of the repeat blocks were identified by Blat [46] to the July 2003 UCSC version h16 [10]. The DNA sequences in between the Blat match limits were fetched from the browser and also analyzed for their similarity to the DEF cluster repeats by Blast and sim.

### Chimpanzee BAC clones

The clones were identified by Blast of the revised alignment consensus to the chimpanzee BAC end sequence database [47]. Subcloning was performed into pUC18 followed by sequencing using dye terminator chemistry and ABI 3730/3700 technology. Base calling and assembly were performed by Phred/Phrap and GAP4 was used for editing and finishing in accordance to the Human Genome Project standards [48].

### Sequence annotation

The gene annotation was performed by using the automated sequence annotation system RUMMAGE [49] and ANA_NOTES, SPANDIT and LACE as a local client of the HAVANA pipeline at the Sanger Institute (Hinxton, UK; [21]. Detailed descriptions of the analysis tools are given by [50] and [51].

### Haplotypes and copy numbers

Genomic DNA was extracted from the blood of four male volunteers using the QIAamp DNA Blood Kit (Qiagen). PCR was performed in a total volume of 25 μl using ReadyToGo PCR beads (Amersham) with 5 pmoles of each primer and 100 ng of DNA. Cycling conditions were 94°C for 30 sec followed by 35 cycles with 94°C for 20 sec, 58°C for 30 sec and 72°C for 60 sec, plus a final 72°C extension for 10 min. Oligos used were for *DEFB4 *GGCGATACTGACACAGGGTT (sense) and ATGGGGAAGGTCAAGGAATC (antisense) and for *DEFB104 *TTCTGTAGCCCCAACACCTC (sense) and GGTGCCAAGGACATCTAGGA (antisense), respectively. PCR products were cloned into PCRTopo2.1 (Invitrogen) and individual clones were sequenced as described above.

## List of abbreviations

BAC Bacterial artificial chromosome

DEF Defensin

FISH Fluorescence *in situ *hybridization

GAP Genome assembly program

HAVANA Human and vertebrate analysis and annotation initiative

LCR Low copy repeat

NCBI National Center for Biotechnology Information

PCR Polymerase chain reaction

ptr Pan troglodytes (chimpanzee)

SNP Single nucleotide polymorphism

UCSC University of California Santa Cruz

WD Working draft

WGS Whole genome shotgun

## Author's contributions

ST performed the assembly revision, the gene annotations, the LCR, SNP and chimpanzee sequence analyses and drafted the manuscript. PG performed mapping and repeat analyses in the 8p23.1 region, completed by IFL's FISH experiments. KR contributed to the sequencing process of clones in the region. AS, SA, NS, KS and MS accounted for the assembly revision and the LCR analyses. AF and RS supported the gene annotations and the DEF structure and function discussions. KH and KR were in charge for the individual haplotype and copy number determinations. MP conceived, designed and coordinated the project.

## Supplementary Material

Additional File 1 Accession numbers, libraries and clone names of all clones shown in main text, Fig. 1. Click here for file

Additional File 3 LCR of clones SCb-561b17 [GenBank:AF238378]; green/yellow signals) and CTB-415D8 [GenBank:AF228730]; red signals) visualized by FISH on metaphase chromosomes according to standard protocols [52,53]. Metaphase spread after DAPI counter stain (A) and color inversion (B). Targeted chromosomes are numbered in B. Any FISH signal is shown quadruplicated within the metaphase spread on four chromatides from two homologue chromosomes. Probe CTB-415D8 generated strong FISH signals with declining intensity at 8p23, 4p16, 11q13.3 and 3q21, corresponding to the in silico identified LCR type II paralogs (see Fig. 3, main text). Additional weaker signals at 3p12-13, 7q21, 11p15, 12p13 and 16p13.3 are also an indication for LCR type II and III paralogs. In contrast, the single locus of SCb-561b17 at 8p23 highlighted by open triangles corresponds to the unique LCR type I. Double signals, resulting from two close located targets at 4p16, 8p23 and 3q21 are marked by asterisks (compare also Additional_file_4). Click here for file

Additional File 4 Resolving LCR type II and III "pairs" on chromosomes at approx. 900 band stage. Probe CTB-415D8 [GenBank:AF228730]; LCR type II and III) generates two clearly separated FISH signals at 4p16 and 8p23, respectively (red signals marked by double arrows): In contrast, probe SCb-561b17 [GenBank:AF238378]; LCR type I) yield a single signal at 8p23, solely (green/yellow signal, open triangle), that is co-localized with the telomeric signal of probe CTB-415D8 [GenBank:AF228730]. Signals with lower intensity are indeterminable in this picture. Click here for file

Additional File 5 DEF aa sequences with highlighted residues (bold) different between human and chimpanzee. Boxes: human aa – human position – chimpanzee aa. All aa positions refer to the following human protein accessions: DEFA6 = [GenBank:NP_001917]; DEFA4 = [GenBank:NP_001916]; DEFA1 = [GenBank:NP_004075]; DEFA5 = [GenBank:NP_066290]; DEFB1 = [GenBank:NP_005209]; DEFB107 = [GenBank:AAM93909]; DEFB105 = [GenBank:NP_689463]; DEFB103 = [GenBank:NP_061131]; DEFB4 = [GenBank:NP_004933]; DEFB108 = [GenBank:AAN33116]. Aa for the chimpanzee orthologs ptrDEFA6, ptrDEFA4, ptrDEFA1, ptr novel defensin similar to DEFA4, ptrDEFA5 and ptrDEFB1 (*ptrDEF cluster a*) are deduced from the chimpanzee WD and might therefore include sequencing errors. Those for ptrDEFB1, ptrDEFB107, ptrDEFB105, ptrDEFB103, ptrDEFB4 and ptrDEFB108 (*DEF cluster b*) are derived from high quality BAC sequences and the appropriate traces were visually inspected. The gray shadow indicates the motif of six cystein residues (except for DEFB107 with only five cysteins). Click here for file

Additional File 2 Synonymous and non synonymous changes by SNPs in human DEF genes and their ancestral alleles by comparison to chimpanzee sequences. Click here for file

Additional File 6 Predicted genomic organization of the human 8p23.1 DEF locus. For simplicity only the DEF clusters (arrows) as well as LCRs type II (rectangles) are shown. *Black*: high quality sequence available; *Gray*: hypothetical structures, no finished sequence available. In addition to a 'minimal' DEF locus consisting of one ***a ***and two ***b ****clusters *(middle), individual loci may have incorporated variable numbers (**F**, **R**) of additional *b clusters *in either orientation. The proposed duplicon consists of two inverted LCRs flanking a *DEF cluster b *(top/bottom). The orientation of any *DEF cluster b *can change either by inverted duplication/crossover (**i**) or homologous recombination within inverted LCRs (**x**, right). Moreover, the proposed genomic structure indicates that even in a 'minimal' DEF locus one or both DEF clusters may be deleted due to homologous recombination between direct LCR copies (Δ). Sequence features of the most distal LCR (II.1; see text and Fig. 3) suggest, that it may be less often involved in recombination or gene conversion events. Click here for file
