# Supplementary material for: Polymorphic segmental duplications at 8p23.1 challenge the determination of individual defensin gene repertoires and the assembly of a contiguous human reference sequence
Source: BMC Genomics. 2004 Dec 10;5:92. doi: 10.1186/1471-2164-5-92 (PMC544879; doi:10.1186/1471-2164-5-92)
Supplement: Additional File 1 — Accession numbers, libraries and clone names of all clones shown in main text, Fig. 1. [file 1471-2164-5-92-S1.pdf]

Additional_file_1

Accession numbers, libraries and clone names of all clones shown in main text, Fig. 1

Number	GenBank accession no.	Library-Clone name
1	AC018398	RP11-16G12
2	AF287957	CTD-2541M15
3	AF233439	GS1-24F4,CTD-2629I16
4	AF200455	SCb-540n10
5	AF238378	SCb-561b17
6	AF228730	SCb-332a23,CTB-415D8
7	AF215847	CTB-415D8
8	AC130339	RP13-941O14
9	AC130360	RP11-287P18
10	AC130367	RP11-1322A13
11	AC134395	RP11-739E3
12	AC134683	RP11-1175L4
13	AF285443	SCb-449o20
14	AF202031	SCb-497j4
15	AC134684	RP11-1118M6
16	AC084121	RP11-623J22
17	AC144950	RP11-191L23
18	AC130365	RP11-1195F20
19	AC131269	RP11-416I21
20	AC105233	RP11-52B19
21	AC068020	RP11-556O5
22	AC068353	RP11-399J23
23	AF298854	SCb-207i3
24	AF205406	SCb-633e22
25	AF314060	GS-i2046
26	AF314059	SCb-540n10
27	AF252831	SCb-177k12
28	AF189745	SCb-324n11
29	AF252830	SCb-295j18
30	AC148106	RP11-65P161
31	AC105214	RP11-354G4
32	AC092766	RP11-483N3
