# Supplementary figures and images for: Polymorphic segmental duplications at 8p23.1 challenge the determination of individual defensin gene repertoires and the assembly of a contiguous human reference sequence
Source: BMC Genomics. 2004 Dec 10;5:92. doi: 10.1186/1471-2164-5-92 (PMC544879; doi:10.1186/1471-2164-5-92)

**A**

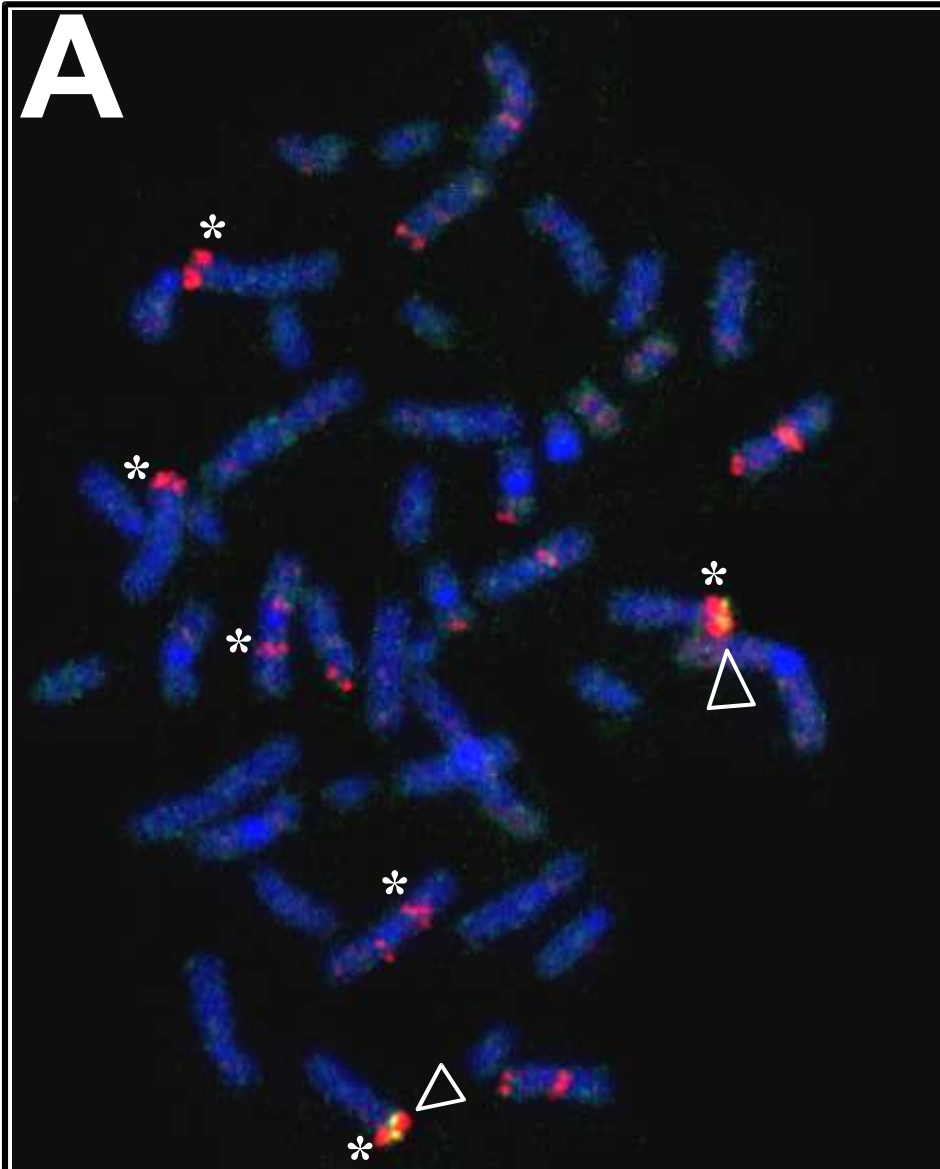

**B**

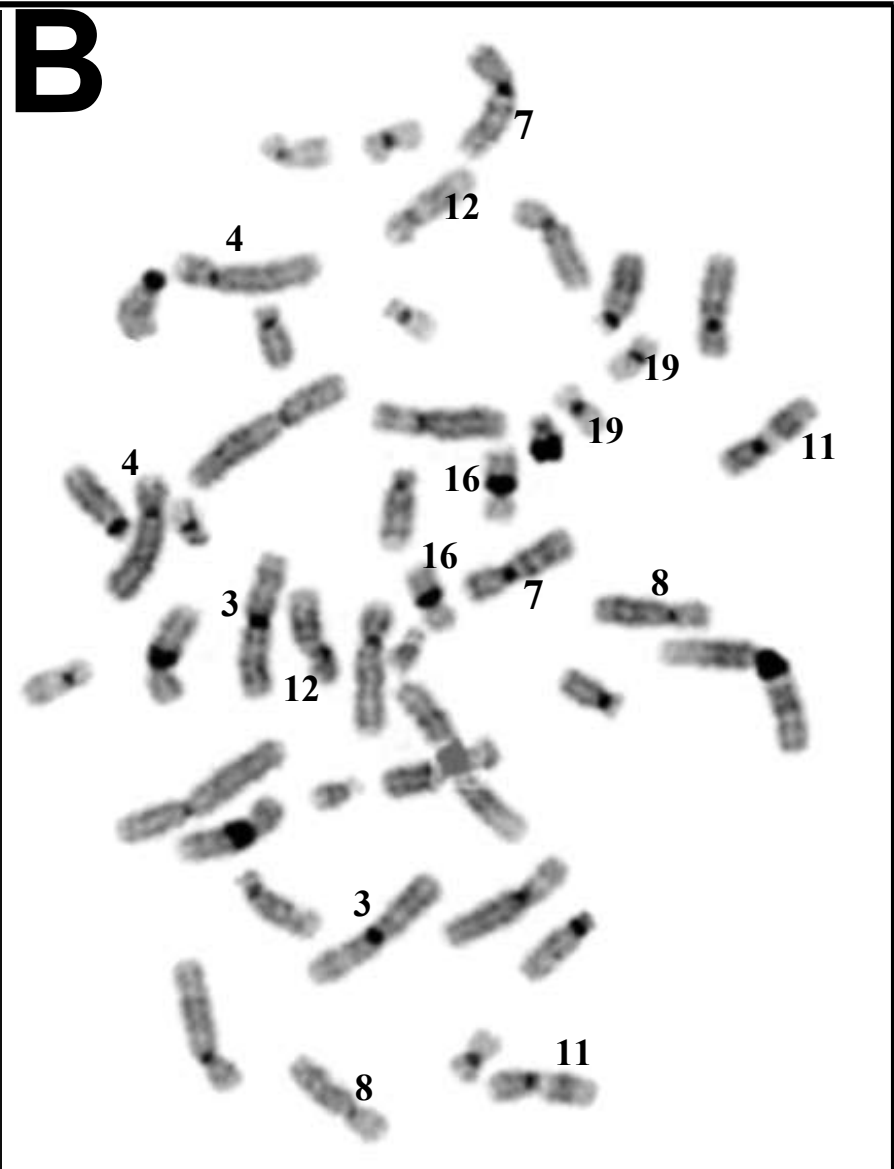

Supplement: Additional File 3 — LCR of clones SCb-561b17 [GenBank:AF238378]; green/yellow signals) and CTB-415D8 [GenBank:AF228730]; red signals) visualized by FISH on metaphase chromosomes according to standard protocols [52,53]. Metaphase spread after DAPI counter stain (A) and color inversion (B). Targeted chromosomes are numbered in B. Any FISH signal is shown quadruplicated within the metaphase spread on four chromatides from two homologue chromosomes. Probe CTB-415D8 generated strong FISH signals with declining intensity at 8p23, 4p16, 11q13.3 and 3q21, corresponding to the in silico identified LCR type II paralogs (see Fig. 3, main text). Additional weaker signals at 3p12-13, 7q21, 11p15, 12p13 and 16p13.3 are also an indication for LCR type II and III paralogs. In contrast, the single locus of SCb-561b17 at 8p23 highlighted by open triangles corresponds to the unique LCR type I. Double signals, resulting from two close located targets at 4p16, 8p23 and 3q21 are marked by asterisks (compare also Additional_file_4). [file 1471-2164-5-92-S3.pdf]

Additional\_file\_4

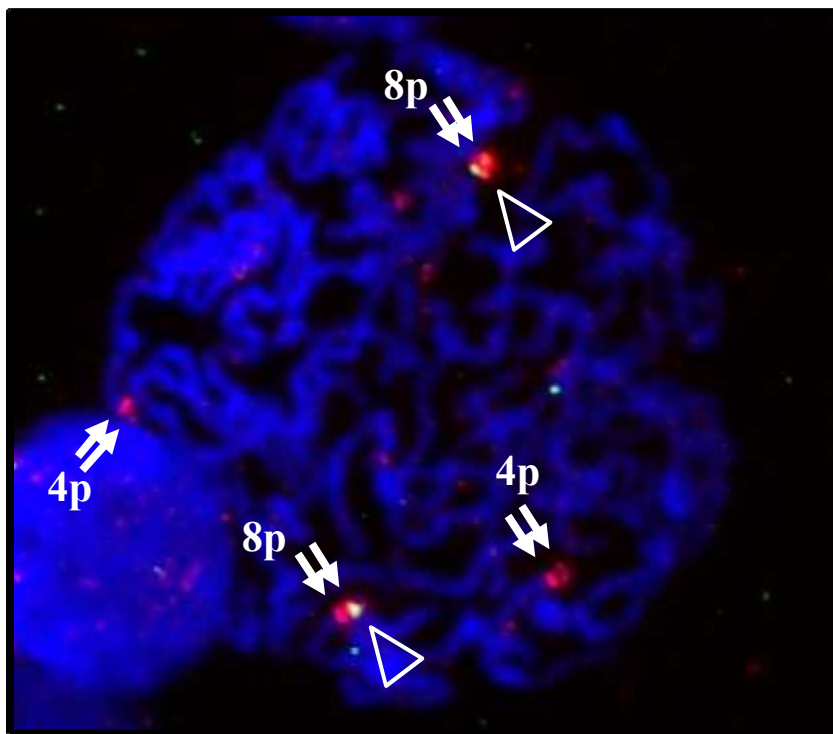

Supplement: Additional File 4 — Resolving LCR type II and III "pairs" on chromosomes at approx. 900 band stage. Probe CTB-415D8 [GenBank:AF228730]; LCR type II and III) generates two clearly separated FISH signals at 4p16 and 8p23, respectively (red signals marked by double arrows): In contrast, probe SCb-561b17 [GenBank:AF238378]; LCR type I) yield a single signal at 8p23, solely (green/yellow signal, open triangle), that is co-localized with the telomeric signal of probe CTB-415D8 [GenBank:AF228730]. Signals with lower intensity are indeterminable in this picture. [file 1471-2164-5-92-S4.pdf]

$\alpha$ -Defensins

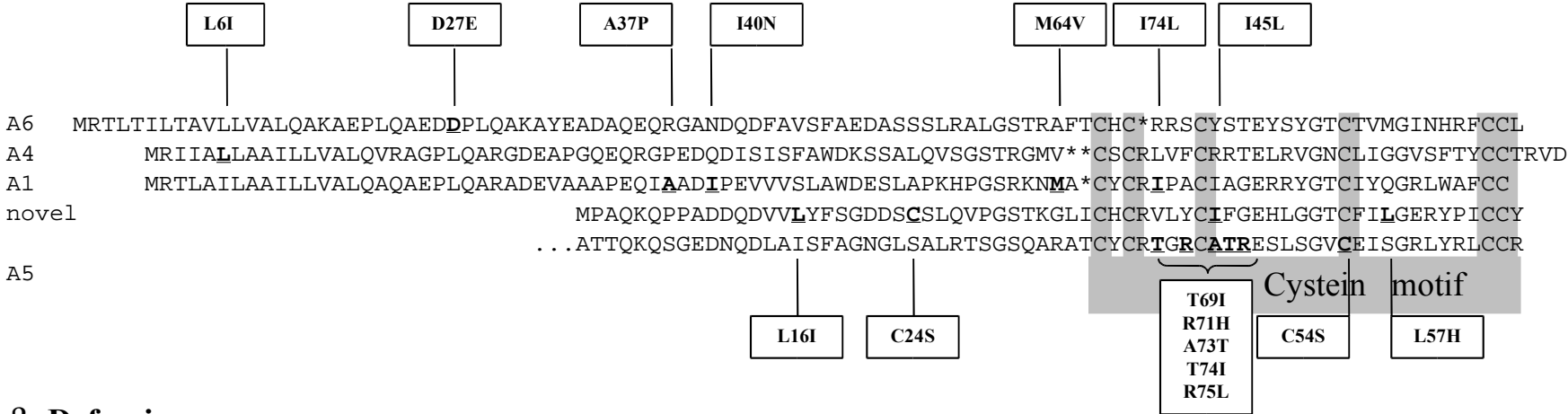

$\beta$ -Defensins

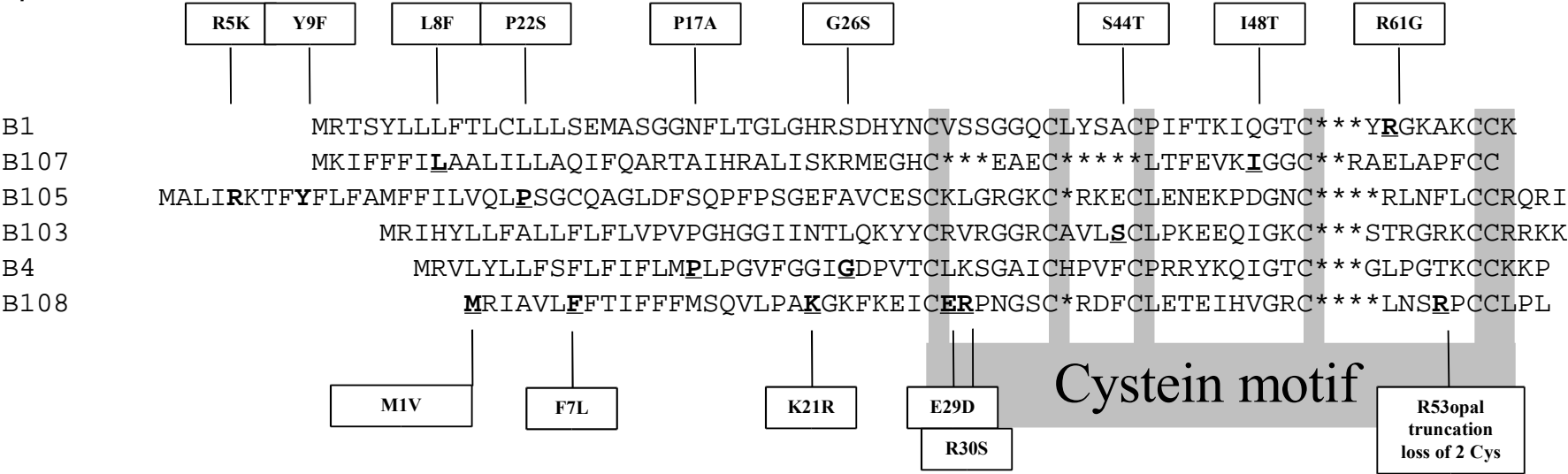

Supplement: Additional File 5 — DEF aa sequences with highlighted residues (bold) different between human and chimpanzee. Boxes: human aa – human position – chimpanzee aa. All aa positions refer to the following human protein accessions: DEFA6 = [GenBank:NP_001917]; DEFA4 = [GenBank:NP_001916]; DEFA1 = [GenBank:NP_004075]; DEFA5 = [GenBank:NP_066290]; DEFB1 = [GenBank:NP_005209]; DEFB107 = [GenBank:AAM93909]; DEFB105 = [GenBank:NP_689463]; DEFB103 = [GenBank:NP_061131]; DEFB4 = [GenBank:NP_004933]; DEFB108 = [GenBank:AAN33116]. Aa for the chimpanzee orthologs ptrDEFA6, ptrDEFA4, ptrDEFA1, ptr novel defensin similar to DEFA4, ptrDEFA5 and ptrDEFB1 (ptrDEF cluster a) are deduced from the chimpanzee WD and might therefore include sequencing errors. Those for ptrDEFB1, ptrDEFB107, ptrDEFB105, ptrDEFB103, ptrDEFB4 and ptrDEFB108 (DEF cluster b) are derived from high quality BAC sequences and the appropriate traces were visually inspected. The gray shadow indicates the motif of six cystein residues (except for DEFB107 with only five cysteins). [file 1471-2164-5-92-S5.pdf]

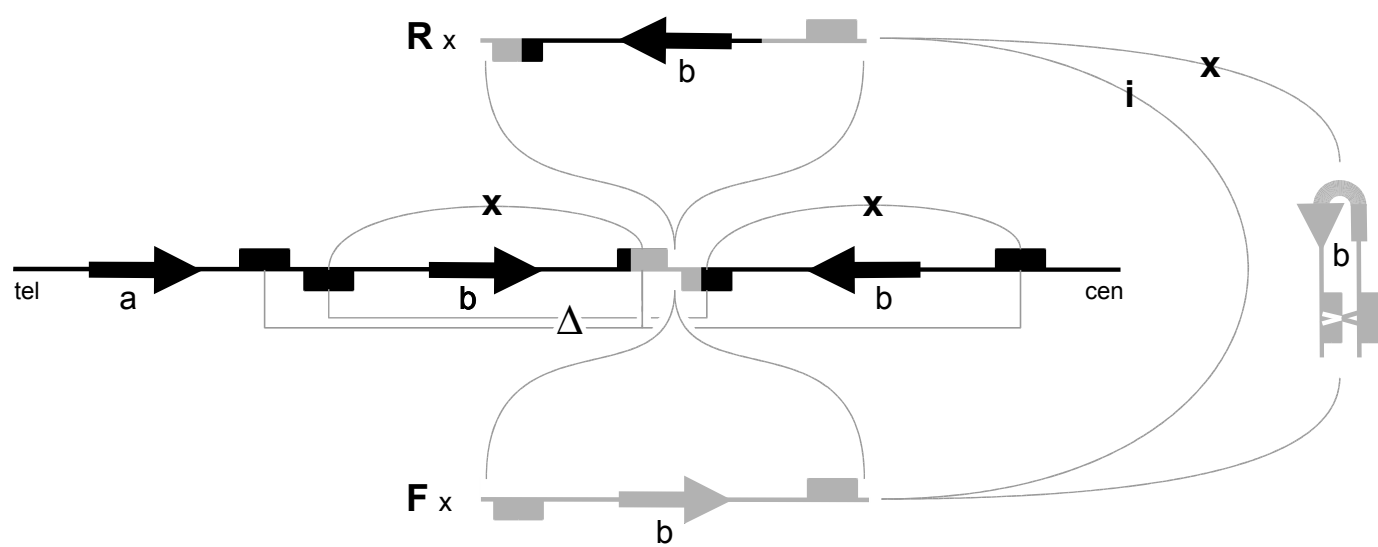

Supplement: Additional File 6 — Predicted genomic organization of the human 8p23.1 DEF locus. For simplicity only the DEF clusters (arrows) as well as LCRs type II (rectangles) are shown. Black: high quality sequence available; Gray: hypothetical structures, no finished sequence available. In addition to a 'minimal' DEF locus consisting of one a and two b clusters (middle), individual loci may have incorporated variable numbers (F, R) of additional b clusters in either orientation. The proposed duplicon consists of two inverted LCRs flanking a DEF cluster b (top/bottom). The orientation of any DEF cluster b can change either by inverted duplication/crossover (i) or homologous recombination within inverted LCRs (x, right). Moreover, the proposed genomic structure indicates that even in a 'minimal' DEF locus one or both DEF clusters may be deleted due to homologous recombination between direct LCR copies (Δ). Sequence features of the most distal LCR (II.1; see text and Fig. 3) suggest, that it may be less often involved in recombination or gene conversion events. [file 1471-2164-5-92-S6.pdf]
