# Supplementary material for: Polymorphic segmental duplications at 8p23.1 challenge the determination of individual defensin gene repertoires and the assembly of a contiguous human reference sequence
Source: BMC Genomics. 2004 Dec 10;5:92. doi: 10.1186/1471-2164-5-92 (PMC544879; doi:10.1186/1471-2164-5-92)
Supplement: Additional File 2 — Synonymous and non synonymous changes by SNPs in human DEF genes and their ancestral alleles by comparison to chimpanzee sequences. [file 1471-2164-5-92-S2.pdf]

Additional_file_2

Synonymous and non synonymous changes by SNPs in human DEF genes and their ancestral alleles by comparison to chimpanzee sequences.

Gene	RefSeq	Base Position	Human SNP entry	alleles	aa change	Chimp ancestral allele
DEFB107	AY122467	13	rs2246582	G/T	F5V	T
DEFB105	NM_152250	107	rs2737995	C/T	P36L	C
DEFB106	NM_152251	125	rs2293957	C/T		T
DEFB104	NM_080389	42	rs11774031	G/A	V10I	A
DEFB4	NM_004942	78		C/T		C
		120	rs2740090	C/T		T
DEFB108	AF540980	97	rs2739903	G/A	G33S	G
		111	rs2740647	C/T		C
		138	rs2719547	A/T		T
DEFB1	NM_005218	152	rs1800957	C/T		C
		160	rs1804747	G/A	H30R	A
		182	rs5743490	C/T		C
		183	rs2738047	G/A	V38I	G
		214	rs1800967	C/T	A48V	C
		270	rs1800968	A/T	C67S	T
DEFA4	NM_001925	306	rs2738100	C/T		T
DEFT1	NM_139127	135	rs1985038	G/A		A
DEFA5	NM_021010	247	rs2272719	C/T		C
		252	rs7839771	G/A	R71H	A
